# Fault-tolerant partition resolvability of cycle with chord

**DOI:** 10.1371/journal.pone.0313300

**Published:** 2024-11-06

**Authors:** Kamran Azhar, Sohail Zafar, Asim Nadeem, Yilun Shang

**Affiliations:** 1 Forman Christian College (A Chartered University), Lahore, Pakistan; 2 University of Management and Technology (UMT), Lahore, Pakistan; 3 Department of Computer and Information Sciences, Northumbria University, Newcastle upon Tyne, United Kingdom; PLOS, UNITED KINGDOM OF GREAT BRITAIN AND NORTHERN IRELAND

## Abstract

In the realm of connected networks, distance-based parameters, particularly the partition dimension of graphs, have extensive applications across various fields, including chemistry and computer science. A notable variant of the partition dimension is the fault-tolerant resolving partition, which is critical in computer science for networking, optimization, and navigation tasks. In networking, fault-tolerant partitioning ensures robust communication pathways even in the event of network failures or disruptions. In optimization, it aids in developing efficient algorithms capable of withstanding errors or changes in input data. In navigation systems, fault-tolerant partitioning supports reliable route planning and navigation services under uncertain or dynamic conditions. This paper focuses on the fault-tolerant partition dimension within the specific context of the cycle with chord graphs, exploring its properties and implications for enhancing the robustness and reliability of networked systems.

## Introduction

Graph theory is a pivotal field of study with profound implications in today’s digital era. It finds extensive applications in robotics, chemistry, and computer science. In particular, graph theory addresses challenges in computer networks, robot network localization, and the development of various chemical network structures. Applications of cycles with chords are notable in areas such as distributed cycle detection [1] and routing optimization [2]. Additionally, this concept is useful in studying diffusion mechanisms and scheduling aircraft. Understanding how to determine vertex positions in a connected graph using distance parameters is crucial for managing graph-structured networks. One significant parameter in this context is the metric dimension (MD) of a graph, which has proven valuable in many scientific domains. The concept of the metric dimension, defined as the minimal cardinality of a resolving set within a graph, was independently introduced by Slater and Harary et al. [3, 4] and recently generalized in various scenarios; see e.g. [5–7].

Chartrand et al. [8] introduced the concept of partition dimension (PD) as a standardized alternative to the metric dimension (MD) of a graph. The PD has been calculated for various classes of graphs. For instance, Khabyah et al. determined the PD of nanosheets and nanotubes derived from octagonal grids [9]. Bhatti et al. examined the PD of generalized hexagonal cellular networks and its applications [10]. Chu et al. addressed the PD problem in the context of convex polytopes [11], while Wei et al. explored cycle-related graphs [12].

Garey et al. [13] highlighted that computing the MD for general graphs is an NP-hard problem. Subsequently, Khuller et al. [14] also confirmed the NP-hardness of the MD problem. Given that the PD is a variant of the MD, computing the PD similarly encounters significant computational complexity, thereby establishing it as an NP-hard problem as well.

Javaid et al. [15] introduced and investigated the concept of fault-tolerant partition dimension (FTPD) in graph theory, representing a significant advancement in the study and application of partition dimension (PD). The FTPD enhances the traditional PD by incorporating resilience against faults or errors in the vertex identification process within a graph. This development is particularly relevant to practical scenarios where robustness and reliability are paramount, such as in network design and fault-tolerant systems. Kamran et al. explored the FTPD in various contexts, including mesh networks [16], cycle-related graphs [17], and chemical graphs [18]. Additionally, Nadeem et al. examined the FTPD of Toeplitz networks and the 2-partition dimension of circulant graphs *C*_*n*_ (1, 2, 3, 4) [19, 20]. In this study, we build upon these foundational works by investigating the FTPD specifically within the context of the cycle with chord graphs.

### Applications

Metric dimension (MD) finds extensive applications in various fields, including network discovery and verification [21], combinatorial optimization [14], image processing [22], and the modeling of chemical substances. The fault-tolerant partition dimension (FTPD) extends these applications to more specialized areas. It is relevant in routing optimization problems [23], supply chain optimization [24], managing water flow in localities [16], and deploying sensors in residential settings [17].

## Preliminaries

Let *G* be a graph with vertex set *V*_*G*_ and edge set *E*_*G*_. For vertices *a*, *b* ∈ *V*_*G*_, the distance between *a* and *b* in *G*, denoted by *d*(*a*, *b*), is the length of the shortest path connecting them. If *L* ⊆ *V*_*G*_, the distance from *a* to *L*, denoted by *d*(*a*, *L*), is defined as *d*(*a*, *L*) = min{*d*(*a*, *x*) ∣ *x* ∈ *L*}.

For a vertex *a* ∈ *V*_*G*_, the open neighborhood *N*(*a*) consists of all vertices adjacent to *a*, that is, *N*(*a*) = {*b* ∈ *V*_*G*_ ∣ *a* is adjacent to *b*}. The closed neighborhood *N*[*a*] includes the open neighborhood along with *a* itself, i.e., *N*[*a*] = *N*(*a*) ∪ {*a*} (see [25]).

Given a set Ω = {*a*_*i*_ ∣ 1 ≤ *i* ≤ *k*} ⊂ *V*_*G*_, the representation of vertex *a* with respect to Ω, denoted by *r*(*a*∣Ω), is the *k*-dimensional vector ${\left(d\left(a,a_{i}\right)\right)}_{i=1}^{k}$, where each component represents the distance from *a* to the vertex *a*_*i*_ in Ω.

Consider *G* to be a connected graph with vertex set *V*(*G*), which can be partitioned into a partition set Ψ. An ordered *q*-partition Ψ is called a resolving partition if the representations of all vertices are unique. The smallest integer *q* for which such a partition exists is known as the partition dimension (PD) of the graph.

The concept of partition dimension has been advanced to the fault-tolerant partition dimension (FTPD) of a graph. For an ordered *q*-partition Ψ to be considered a fault-tolerant resolving partition, it must ensure that the representations of each pair of distinct vertices in *G* are distinct in at least two positions. The smallest integer *q* for which such a partition exists is referred to as the fault-tolerant partition dimension of the graph, denoted by P(G).

### Important results of FTPD

Below are some notable results concerning the fault-tolerant partition dimension (FTPD) of a connected graph *G* with *n* ≥ 2 vertices.

**Proposition 0.1** [26] 3≤P(G)≤n, *where, n* ≥ 3.

**Proposition 0.2** [25] $P(P_{n})=3$, *where, n* ≥ 3.

**Proposition 0.3** [26] $P(K_{n})=n$ *if and only if G* ≅ *K*_*n*_ *or G* ≅ *K*_*n*_ − *e, where K*_*n*_ *is a complete graph*.

In the remaining part of the article, the FTPD of cycle with chord section is dedicated to computing $P(C_{n}^{t})$, where $C_{n}^{t}$ denotes a cycle with chord graph. The paper concludes in the Conclusion section with the proposal of an open problem.

## FTPD of cycle with chord

**Fig 1:**
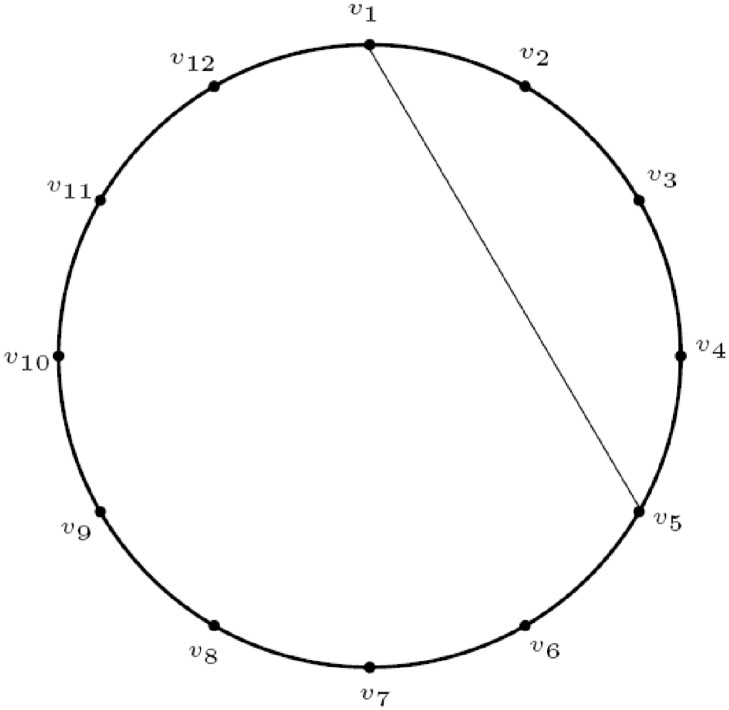
The graph $C_{12}^{4}$.

This part is devoted to the calculation of FTPD of the cycle with chord. The graph $C_{n}^{t}$ is a cycle with a chord, where two vertices at a distance *t* in the cycle *C*_*n*_ are connected by an edge. The $V\left(C_{n}^{t}\right)=\left\{v_{q}:1\leq q\leq n\right\}$ and $E\left(C_{n}^{t}\right)=\left\{v_{q}v_{q+1}:1\leq q\leq n-1\right\}\cup \left\{v_{1}v_{n}\right\}\cup \left\{v_{1}v_{t+1}\right\}$ are vertex set and edge set respectively. Clearly, $C_{n}^{t}$ is same as $C_{n}^{n-t}$ for *n* ≥ 4, and 2 ≤ *t* ≤ *n* − 2. Therefore, it is enough to compute $P(C_{n}^{t})$, for *n* ≥ 4 and $2\leq t\leq \lfloor \frac{n}{2}\rfloor$. Graph of cycle with chord $C_{12}^{4}$ is shown in Fig 1.

**Lemma 0.4** [27] *For n* ≥ 4 *and* $2\leq t\leq \lfloor \frac{n}{2}\rfloor$, *MD of* $C_{n}^{t}=2$.

**Lemma 0.5** [12] *For n* ≥ 4 *and* $2\leq t\leq \lfloor \frac{n}{2}\rfloor$, *PD of* $C_{n}^{t}=3$.

Following is the main result of the paper which computes the FT partition dimension of $C_{n}^{t}$.

**Theorem 0.6** *For n* ≥ 4 *and* $2\leq t\leq \lfloor \frac{n}{2}\rfloor$, $P(C_{n}^{t})=4$.

**Proof.** To demonstrate that $P(C_{n}^{t})=4$, we first establish $P(C_{n}^{t})\leq 4$. In this context, consider Ψ = {Ψ_1_, Ψ_2_, Ψ_3_, Ψ_4_} be a partition of $V(C_{n}^{t})$, next, we examine the following cases:

**Case (i) For** 4 ≤ *n* ≤ 5.
  It is evident that Ψ forms FT resolving partition of $C_{n}^{t}$, with Ψ_1_ = {*v*_1_}, Ψ_2_ = {*v*_2_}, Ψ_3_ = {*v*_3_} and Ψ_4_ = {*v*_*q*_ : 4 ≤ *q* ≤ *n*}.
**Case (ii) For** *n* ≥ 6, we have the following subcases:
**Subcase ii(a)** If *n* = 2*ζ* and 2 ≤ *t* ≤ *ζ* − 1, then representation of vertices (*R*_*v*_) with regard to Ψ_1_ = {*v*_*q*_ : 1 ≤ *q* ≤ *ζ*}, Ψ_2_ = {*v*_*ζ*+1_}, Ψ_3_ = {*v*_*q*_ : *ζ* + 2 ≤ *q* ≤ *ζ* + *t*} and Ψ_4_ = {*v*_*q*_ : *ζ* + *t* + 1 ≤ *q* ≤ *n*} can be seen in Tables 1–6.
**Subcase ii(b)** If *n* = 2*ζ* and *t* = *ζ*, then *R*_*v*_ with regard to Ψ_1_ = {*v*_*q*_ : 1 ≤ *q* ≤ *ζ* − 1}, Ψ_2_ = {*v*_*ζ*_}, Ψ_3_ = {*v*_*q*_ : *ζ* + 1 ≤ *q* ≤ *n* − 1} and Ψ_4_ = {*v*_*n*_} can be seen in Tables 7 and 8.
**Subcase ii(c)** If *n* = 2*ζ* + 1 and 2 ≤ *t* ≤ *ζ* − 1, then *R*_*v*_ with regard to Ψ_1_ = {*v*_*q*_ : 1 ≤ *q* ≤ *ζ*}, Ψ_2_ = {*v*_*ζ*+1_}, Ψ_3_ = {*v*_*q*_ : *ζ* + 2 ≤ *q* ≤ *ζ* + *t* + 1} and Ψ_4_ = {*v*_*q*_ : *ζ* + *t* + 2 ≤ *q* ≤ *n*} can be seen in Tables 9–14.
**Subcase ii(d)** If *n* = 2*ζ* + 1 and *t* = *ζ*, then *R*_*v*_ with regard to Ψ_1_ = {*v*_*q*_ : 1 ≤ *q* ≤ *ζ* + 1}, Ψ_2_ = {*v*_*ζ*+2_}, Ψ_3_ = {*v*_*q*_ : *ζ* + 3 ≤ *q* ≤ *n* − 1} and Ψ_4_ = {*v*_*n*_} can be seen in Tables 15 and 16.
  It is clear from Cases (i)-(ii), that Ψ is FT resolving partition of $C_{n}^{t}$, therefore, $P(C_{n}^{t})\leq 4$.

**Table 1:** *R*_*v*_ for *n* = 2*ζ* and *t* = 2*ϖ* − 1 where $2\leq \varpi \leq \;\lfloor \frac{\zeta -1}{4}\rfloor +1$.

Distance of vertices from	Ψ_1_	Ψ_2_	Ψ_3_	Ψ_4_
*v*_*χ*_(1 ≤ *χ* ≤ *ϖ*)	0	*ζ* − 2*ϖ* + *χ* + 1	*ζ* − 2*ϖ* + *χ* + 1	*χ*
*v*_*ϖ*+*χ*_(1 ≤ *χ* ≤ *ϖ*)	0	*ζ* − *ϖ* − *χ* + 1	*ζ* − *ϖ* − *χ* + 2	*ϖ* − *χ* + 2
$v_{2\varpi +\chi -2}\left(3\leq \chi \leq \;\left\lfloor \frac{\zeta }{2}\right\rfloor +1\right)$	0	*ζ* − 2*ϖ* − *χ* + 3	*ζ* − 2*ϖ* − *χ* + 4	*χ*
$v_{\zeta -\chi +1}\left(1\leq \chi \leq \;\left\lceil \frac{\zeta }{2}\right\rceil -2\varpi +1\right)$	0	*χ*	*χ* + 1	2*ϖ* + *χ* − 1
*v*_*ζ*+1_	1	0	1	2*ϖ* − 1
*v*_*ζ*+*χ*_(2 ≤ *χ* ≤ 2*ϖ* − 1)	*χ*	*χ* − 1	0	2*ϖ* − *χ*
$v_{\zeta +2\varpi +\chi -1}\left(1\leq \chi \leq \;\left\lceil \frac{\zeta }{2}\right\rceil -2\varpi +1\right)$	2*ϖ* + *χ* − 1	2*ϖ* + *χ* − 2	*χ*	0
$v_{2\zeta -\chi +1}\left(\varpi -1\leq \chi \leq \;\left\lfloor \frac{\zeta }{2}\right\rfloor \right)$	*χ*	*ζ* − *χ*	*ζ* − *χ* − 2*ϖ* + 2	0
*v*_2*ζ*−*ϖ*+*χ*+2_(1 ≤ *χ* ≤ *ϖ* − 1)	*ϖ* − *χ* − 1	*ζ* − *ϖ* − *χ* + 1	*ζ* − 3*ϖ* + *χ* + 3	0

**Table 2:** *R*_*v*_ for *n* = 2*ζ* and *t* = 2*ϖ* − 1. For 8 ≤ *n* ≤ 36, $\varpi =\lfloor \frac{\zeta -1}{4}\rfloor +2$ and for *n* ≥ 38, $\left\lfloor \frac{\zeta -1}{4}\right\rfloor +2\leq \varpi \leq \left\lfloor \frac{\zeta -1}{4}\right\rfloor +3$.

Distance of vertices from	Ψ_1_	Ψ_2_	Ψ_3_	Ψ_4_
*v*_*χ*_(1 ≤ *χ* ≤ *ϖ*)	0	*ζ* − 2*ϖ* + *χ* + 1	*ζ* − 2*ϖ* + *χ* + 1	*χ*
*v*_*ϖ*+*χ*_(1 ≤ *χ* ≤ *ϖ*)	0	*ζ* − *ϖ* − *χ* + 1	*ζ* − *ϖ* − *χ* + 2	*ϖ* − *χ* + 2
*v*_2*ϖ*+*χ*−2_(3 ≤ *χ* ≤ *ζ* − 2*ϖ* + 2)	0	*ζ* − 2*ϖ* − *χ* + 3	*ζ* − 2*ϖ* − *χ* + 4	*χ*
*v*_*ζ*+1_	1	0	1	*ζ* − 2*ϖ* + 3
$v_{2\varpi +\chi -2}\left(\zeta -2\varpi +4\leq \chi \leq \;\left\lfloor \frac{\zeta }{2}\right\rfloor +1\right)$	*χ* − *ζ* + 2*ϖ* − 2	*χ* − *ζ* + 2*ϖ* − 3	0	*χ*
$v_{\zeta +\chi }\left(2\varpi -\left\lceil \frac{\zeta }{2}\right\rceil \leq \chi \leq \;\left\lceil \frac{\zeta }{2}\right\rceil \right)$	*χ*	*χ* − 1	0	2*ϖ* − *χ*
$v_{\zeta +2\varpi -\chi }\left(1\leq \chi \leq \;2\varpi -\left\lceil \frac{\zeta }{2}\right\rceil -1\right)$	*h* − 2*ϖ* + *χ* + 1	2*ϖ* − *χ* − 1	0	*χ*
*v*_*ζ*+2*ϖ*+*χ*−1_(1 ≤ *χ* ≤ *ζ* − 3*ϖ* + 3)	*ζ* − 2*ϖ* − *χ* + 2	2*ϖ* + *χ* − 2	*χ*	0
*v*_2*ζ*−*χ*+1_(1 ≤ *χ* ≤ *ϖ* − 2)	*χ*	*ζ* − 2*ϖ* + *χ* + 2	*ζ* − 2*ϖ* − *χ* + 2	0

**Table 3:** *R*_*v*_ for *n* = 2*ζ* and *t* = 2*ϖ* − 1. For 16 ≤ *n* ≤ 36, $\left\lfloor \frac{\zeta -1}{4}\right\rfloor +3\leq \varpi \leq \left\lfloor \frac{\zeta }{2}\right\rfloor$ and for *n* ≥ 38, $\left\lfloor \frac{\zeta -1}{4}\right\rfloor +4\leq \varpi \leq \left\lfloor \frac{\zeta }{2}\right\rfloor$.

Distance of vertices from	Ψ_1_	Ψ_2_	Ψ_3_	Ψ_4_
*v*_*χ*_(1 ≤ *χ* ≤ *ϖ*)	0	*ζ* − 2*ϖ* + *χ* + 1	*ζ* − 2*ϖ* + *χ* + 1	*χ*
*v*_*ϖ*+*χ*_(1 ≤ *χ* ≤ *ϖ*)	0	*h* − *ϖ* − *χ* + 1	*ζ* − *ϖ* − *χ* + 2	*ϖ* − *χ* + 2
*v*_2*ϖ*+*χ*−2_(3 ≤ *χ* ≤ *ζ* − 2*ϖ* + 2)	0	*ζ* − 2*ϖ* − *χ* + 3	*ζ* − 2*ϖ* − *χ* + 4	*χ*
*v*_*ζ*+1_	1	0	1	*ζ* − 2*ϖ* + 3
$v_{2\varpi +\chi -2}\left(\zeta -2\varpi +4\leq \chi \leq \;\left\lfloor \frac{\zeta }{2}\right\rfloor +1\right)$	*χ* − *ζ* + 2*ϖ* − 2	*χ* − *ζ* + 2*ϖ* − 3	0	*χ*
$v_{\zeta +\chi }\left(2\varpi -\left\lceil \frac{\zeta }{2}\right\rceil \leq \chi \leq \;\left\lceil \frac{\zeta }{2}\right\rceil \right)$	*χ*	*χ* − 1	0	2*ϖ* − *χ*
$v_{2\zeta -\chi +1}\left(\varpi -1\leq \chi \leq \;\left\lfloor \frac{\zeta }{2}\right\rfloor \right)$	*χ*	*ζ* − *χ*	0	2*ϖ* − *ζ* + *χ* − 1
*v*_*ζ*+2*ϖ*−*χ*_(1 ≤ *χ* ≤ 3*ϖ* − *ζ* − 3)	*ζ* − 2*ϖ* + *χ* + 1	2*ζ* − 4*ϖ* + *χ* + 3	0	*χ*
*v*_2*ζ*−*χ*+1_(1 ≤ *χ* ≤ *ζ* − 2*ϖ* + 1)	*χ*	*ζ* − 2*ϖ* + *χ* + 2	*ζ* − 2*ϖ* − *χ* + 2	0

**Table 4:** *R*_*v*_ for *n* = 2*ζ* and *t* = 2*ϖ* where $1\leq \varpi \leq \;\lfloor \frac{\zeta +1}{4}\rfloor$.

Distance of vertices from	Ψ_1_	Ψ_2_	Ψ_3_	Ψ_4_
*v*_*χ*_(1 ≤ *χ* ≤ *ϖ*)	0	*ζ* − 2*ϖ* + *χ*	*ζ* − 2*ϖ* + *χ*	*χ*
*v*_*ϖ*+1_	0	*ζ* − *ϖ*	*ζ* − *ϖ* + 1	*ϖ* + 1
*v*_*ϖ*+*χ*_(2 ≤ *χ* ≤ *ϖ* + 1)	0	*ζ* − *ϖ* − *χ* + 1	*ζ* − *ϖ* − *χ* + 2	*ϖ* − *χ* + 3
$v_{2\varpi +\chi -1}\left(3\leq \chi \leq \;\left\lfloor \frac{\zeta }{2}\right\rfloor +1\right)$	0	*ζ* − 2*ϖ* − *χ* + 2	*ζ* − 2*ϖ* − *χ* + 3	*χ*
$v_{\zeta -\chi +1}\left(1\leq \chi \leq \;\left\lceil \frac{\zeta }{2}\right\rceil -2\varpi \right)$	0	*χ*	*χ* + 1	2*ϖ* + *χ*
*v*_*ζ*+1_	1	0	1	2*ϖ*
*v*_*ζ*+*χ*_(2 ≤ *χ* ≤ 2*ϖ*)	*χ*	*χ* − 1	0	2*ϖ* − *χ* + 1
$v_{\zeta +2\varpi +\chi }\left(1\leq \chi \leq \;\left\lceil \frac{\zeta }{2}\right\rceil -2\varpi \right)$	2*ϖ* + *χ*	2*ϖ* + *χ* − 1	*χ*	0
$v_{2\zeta -\chi +1}\left(\varpi \leq \chi \leq \;\left\lfloor \frac{\zeta }{2}\right\rfloor \right)$	*χ*	*ζ* − *χ*	*ζ* − *χ* − 2*ϖ* + 1	0
*v*_2*ζ*−*ϖ*+*χ*+1_(1 ≤ *χ* ≤ *ϖ* − 1)	*ϖ* − *χ*	*ζ* − *ϖ* − *χ* + 1	*ζ* − 3*ϖ* + *χ* + 1	0

**Table 5:** *R*_*v*_ for *n* = 2*ζ* and *t* = 2*ϖ*. For 10 ≤ *n* ≤ 24, $\varpi =\lfloor \frac{\zeta +1}{4}\rfloor +1$ and for *n* ≥ 26, $\left\lfloor \frac{\zeta +1}{4}\right\rfloor +1\leq \varpi \leq \left\lfloor \frac{\zeta +1}{4}\right\rfloor +2$.

Distance of vertices from	Ψ_1_	Ψ_2_	Ψ_3_	Ψ_4_
*v*_*χ*_(1 ≤ *χ* ≤ *ϖ*)	0	*ζ* − 2*ϖ* + *χ*	*ζ* − 2*ϖ* + *χ*	*χ*
*v*_*ϖ*+1_	0	*ζ* − *ϖ*	*ζ* − *ϖ* + 1	*ϖ* + 1
*v*_*ϖ*+*χ*+1_(1 ≤ *χ* ≤ *ϖ*)	0	*ζ* − *ϖ* − *χ*	*ζ* − *ϖ* − *χ* + 1	*ϖ* − *χ* + 2
*v*_2*ϖ*+*χ*−1_(3 ≤ *χ* ≤ *ζ* − 2*ϖ* + 1)	0	*ζ* − 2*ϖ* − *χ* + 2	*ζ* − 2*ϖ* − *χ* + 3	*χ*
*v*_*ζ*+1_	1	0	1	*ζ* − 2*ϖ* + 2
$v_{2\varpi +\chi -1}\left(h-2\varpi +3\leq \chi \leq \;\left\lfloor \frac{\zeta }{2}\right\rfloor +1\right)$	*χ* − *ζ* + 2*ϖ* − 1	*χ* − *ζ* + 2*ϖ* − 2	0	*χ*
$v_{\zeta +\chi }\left(2\varpi -\left\lceil \frac{\zeta }{2}\right\rceil +1\leq \chi \leq \;\left\lceil \frac{\zeta }{2}\right\rceil \right)$	*χ*	*χ* − 1	0	2*ϖ* − *χ* + 1
*v*_12_ for *n* = 14	3	4	0	2
*v*_13_ for *n* = 14	2	4	0	1
*v*_14_ for *n* = 14	1	3	1	0
$v_{\zeta +2\varpi -\chi }\left(1\leq \chi \leq \;2\varpi -\left\lceil \frac{\zeta }{2}\right\rceil \right)$ for *n* ≠ 14	*ζ* − 2*ϖ* + *χ*	2*ϖ* − *χ*	0	*χ*
*v*_*ζ*+2*ϖ*+*χ*_(1 ≤ *χ* ≤ *ζ* − 3*ϖ* + 1) for *n* ≠ 14	*ζ* − 2*ϖ* − *χ* + 1	2*ϖ* + *χ* − 1	*χ*	0
*v*_2*ζ*−*χ*+1_(1 ≤ *χ* ≤ *ϖ* − 1) for *n* ≠ 14	*χ*	*ζ* − 2*ϖ* + *χ* + 1	*ζ* − 2*ϖ* − *χ* + 1	0

**Table 6:** *R*_*v*_ for *n* = 2*ζ* and *t* = 2*ϖ*. For 18 ≤ *n* ≤ 24, $\left\lfloor \frac{\zeta +1}{4}\right\rfloor +2\leq \varpi \leq \left\lfloor \frac{2\zeta -1}{4}\right\rfloor$, and for *n* ≥ 26, $\left\lfloor \frac{\zeta +1}{4}\right\rfloor +3\leq \varpi \leq \left\lfloor \frac{2\zeta -1}{4}\right\rfloor$.

Distance of vertices from	Ψ_1_	Ψ_2_	Ψ_3_	Ψ_4_
*v*_*χ*_(1 ≤ *χ* ≤ *ϖ*)	0	*ζ* − 2*ϖ* + *χ*	*ζ* − 2*ϖ* + *χ*	*χ*
*v*_*ϖ*+1_	0	*ζ* − *ϖ*	*ζ* − *ϖ* + 1	*ϖ* + 1
*v*_*ϖ*+*χ*+1_(1 ≤ *χ* ≤ *ϖ*)	0	*ζ* − *ϖ* − *χ*	*ζ* − *ϖ* − *χ* + 1	*ϖ* − *χ* + 2
*v*_2*ϖ*+*χ*−1_(3 ≤ *χ* ≤ *ζ* − 2*ϖ* + 1)	0	*ζ* − 2*ϖ* − *χ* + 2	*ζ* − 2*ϖ* − *χ* + 3	*χ*
*v*_*ζ*+1_	1	0	1	*ζ* − 2*ϖ* + 2
$v_{2\varpi +\chi -1}\left(h-2\varpi +3\leq \chi \leq \;\left\lfloor \frac{\zeta }{2}\right\rfloor +1\right)$	*χ* − *ζ* + 2*ϖ* − 1	*χ* − *ζ* + 2*ϖ* − 2	0	*χ*
$v_{\zeta +\chi }\left(2\varpi -\left\lceil \frac{\zeta }{2}\right\rceil +1\leq \chi \leq \;\left\lceil \frac{\zeta }{2}\right\rceil \right)$	*χ*	*χ* − 1	0	2*ϖ* − *χ* + 1
$v_{2\zeta -\chi +1}\left(\varpi \leq \chi \leq \;\left\lfloor \frac{\zeta }{2}\right\rfloor \right)$	*χ*	*ζ* − *χ*	0	2*ϖ* − *ζ* + *χ*
*v*_*ζ*+2*ϖ*−*χ*+1_(1 ≤ *χ* ≤ 3*ϖ* − *ζ* − 1)	*ζ* − 2*ϖ* + *χ* + 1	2*ζ* − 4*ϖ* + *χ* + 1	0	*χ*
*v*_2*ζ*−*χ*+1_(1 ≤ *χ* ≤ *ζ* − 2*ϖ*)	*χ*	*ζ* − 2*ϖ* + *χ* + 1	*ζ* − 2*ϖ* − *χ* + 1	0

**Table 7:** *R*_*v*_ for *n* = 2*ζ* and *t* = *ζ* = 2*ϖ* − 1.

Distance of vertices from	Ψ_1_	Ψ_2_	Ψ_3_	Ψ_4_
*v*_*χ*_(1 ≤ *χ* ≤ *ϖ* − 1)	0	*χ* + 1	*χ*	*χ*
*v*_*ϖ*+*χ*−1_(1 ≤ *χ* ≤ 2)	0	*h* − *ϖ* − *χ* + 1	*ζ* − *ϖ* − *χ* + 2	*ζ* − *ϖ* + *χ*
*v*_*ϖ*+*χ*+1_(1 ≤ *χ* ≤ *ϖ* − 2)	0	*ζ* − *ϖ* − *χ* − 1	*ζ* − *ϖ* − *χ*	*ζ* − *ϖ* − *χ* + 2
*v*_*ζ*_	1	0	1	3
*v*_*ζ*+*χ*_(1 ≤ *χ* ≤ *ϖ* − 1)	*χ*	*χ*	0	*χ* + 1
*v*_*ζ*+*ϖ*_	*ζ* − *ϖ* − *χ* + 1	*ζ* − *ϖ* + *χ*	0	*ζ* − *ϖ* − *χ*
*v*_*ζ*+*ϖ*+*χ*+1_(1 ≤ *χ* ≤ *ϖ* − 3)	*ζ* − *ϖ* − *χ*	*ζ* − *ϖ* − *χ* + 2	0	*ζ* − *ϖ* − *χ* − 1
*v*_2*ζ*_	1	3	1	0

**Table 8:** *R*_*v*_ for *n* = 2*ζ* and *t* = *ζ* = 2*ϖ*.

Distance of vertices from	Ψ_1_	Ψ_2_	Ψ_3_	Ψ_4_
*v*_*χ*_(1 ≤ *χ* ≤ *ϖ* − 1)	0	*χ* + 1	*χ*	*χ*
*v*_*ϖ*+1_	0	*ϖ*	*ϖ*	*ϖ*
*v*_*ϖ*+2_	0	*ϖ* − 1	*ϖ*	*ϖ* + 1
*v*_*ϖ*+*χ*+1_(1 ≤ *χ* ≤ *ϖ* − 2)	0	*ζ* − *ϖ* − *χ* − 1	*ζ* − *ϖ* − *χ*	*ζ* − *ϖ* − *χ* + 2
*v*_*ζ*_	1	0	1	3
*v*_*ζ*+*χ*_(1 ≤ *χ* ≤ *ϖ* − 1)	*χ*	*χ*	0	*χ* + 1
*v*_*ζ*+*ϖ*_	*ϖ*	*ϖ*	0	*ϖ*
*v*_*ζ*+*ϖ*+1_	*ϖ*	*ϖ* + 1	0	*ϖ* − 1
*v*_*ζ*+*ϖ*+*χ*+1_(1 ≤ *χ* ≤ *ϖ* − 2)	*ζ* − *ϖ* − *χ*	*ζ* − *ϖ* − *χ* + 2	0	*ζ* − *ϖ* − *χ* − 1
*v*_2*ζ*_	1	3	1	0

**Table 9:** *R*_*v*_ for *n* = 2*ζ* + 1 and *t* = 2*ϖ* where $1\leq \varpi \leq \lfloor \frac{\zeta }{4}\rfloor$.

Distance of vertices from	Ψ_1_	Ψ_2_	Ψ_3_	Ψ_4_
*v*_*χ*_(1 ≤ *χ* ≤ *ϖ*)	0	*ζ* − 2*ϖ* + *χ*	*ζ* − 2*ϖ* + *χ*	*χ*
*v*_*ϖ*+1_	0	*ζ* − *ϖ*	*ζ* − *ϖ* + 1	*ϖ* + 1
*v*_*ϖ*+*χ*+1_(1 ≤ *χ* ≤ *ϖ*)	0	*ζ* − *ϖ* − *χ*	*ζ* − *ϖ* − *χ* + 1	*ϖ* − *χ* + 2
$v_{2\varpi +\chi -1}\left(3\leq \chi \leq \left\lceil \frac{\zeta }{2}\right\rceil +1\right)$	0	*ζ* − 2*ϖ* − *χ* + 2	*ζ* − 2*ϖ* − *χ* + 3	*χ*
$v_{\zeta -\chi +1}\left(1\leq \chi \leq \left\lfloor \frac{\zeta }{2}\right\rfloor -2\varpi \right)$	0	*χ*	*χ* + 1	2*ϖ* + *χ* + 1
*v*_*ζ*+1_	1	0	1	2*ϖ* + 1
*v*_*ζ*+*χ*+1_(1 ≤ *χ* ≤ 2*ϖ*)	*χ* + 1	*χ*	0	2*ϖ* − *χ* + 1
$v_{\zeta +2\varpi +\chi +1}\left(1\leq \chi \leq \left\lfloor \frac{\zeta }{2}\right\rfloor -2\varpi \right)$	2*ϖ* + *χ* + 1	2*ϖ* + *χ*	*χ*	0
$v_{2\zeta -\chi +2}\left(\varpi \leq \chi \leq \left\lceil \frac{\zeta }{2}\right\rceil \right)$	*χ*	*ζ* − *χ* + 1	*ζ* − *χ* − 2*ϖ* + 1	0
*v*_2*ζ*−*ϖ*+*χ*+2_(1 ≤ *χ* ≤ *ϖ* − 1)	*ϖ* − *χ*	*ζ* − *ϖ* − *χ* + 1	*ζ* − 3*ϖ* + *χ* + 1	0

**Table 10:** *R*_*v*_ for *n* = 2*ζ* + 1 and *t* = 2*ϖ*. For 7 ≤ *n* ≤ 21, $\varpi =\lfloor \frac{\zeta }{4}\rfloor +1$ and for *n* ≥ 23, $\left\lfloor \frac{\zeta }{4}\right\rfloor +1\leq \varpi \leq \left\lfloor \frac{\zeta }{4}\right\rfloor +2$.

Distance of vertices from	Ψ_1_	Ψ_2_	Ψ_3_	Ψ_4_
*v*_*χ*_(1 ≤ *χ* ≤ *ϖ*)	0	*ζ* − 2*ϖ* + *χ*	*ζ* − 2*ϖ* + *χ*	*χ*
*v*_*ϖ*+1_	0	*ζ* − *ϖ*	*ζ* − *ϖ* + 1	*ϖ* + 1
*v*_*ϖ*+*χ*+1_(1 ≤ *χ* ≤ *ϖ*)	0	*ζ* − *ϖ* − *χ*	*ζ* − *ϖ* − *χ* + 1	*ϖ* − *χ* + 2
*v*_2*ϖ*+*χ*−1_(3 ≤ *χ* ≤ *ζ* − 2*ϖ* + 1)	0	*ζ* − 2*ϖ* − *χ* + 2	*ζ* − 2*ϖ* − *χ* + 3	*χ*
*v*_*ζ*+1_	1	0	1	*ζ* − 2*ϖ* + 2
$v_{2\varpi +\chi -1}\left(h-2\varpi +3\leq \chi \leq \;\left\lceil \frac{\zeta }{2}\right\rceil +1\right)$	*χ* − *ζ* + 2*ϖ* − 1	*χ* − *ζ* + 2*ϖ* − 2	0	*χ*
$v_{\zeta +\chi }\left(2\varpi -\left\lfloor \frac{\zeta }{2}\right\rfloor +1\leq \chi \leq \;\left\lfloor \frac{\zeta }{2}\right\rfloor +1\right)$	*χ*	*χ* − 1	0	2*ϖ* − *χ* + 2
$v_{\zeta +2\varpi -\chi +2}\left(1\leq \chi \leq \;2\varpi -\left\lfloor \frac{\zeta }{2}\right\rfloor \right)$	*ζ* − 2*ϖ* + *χ*	2*ϖ* − *χ* + 1	0	*χ*
*v*_*ζ*+2*ϖ*+*χ*+1_(1 ≤ *χ* ≤ *ζ* − 3*ϖ* + 1)	*ζ* − 2*ϖ* − *χ* + 1	2*ϖ* + *χ*	*χ*	0
*v*_2*ζ*−*χ*+2_(1 ≤ *χ* ≤ *ϖ* − 1)	*χ*	*ζ* − 2*ϖ* + *χ* + 1	*ζ* − 2*ϖ* − *χ* + 1	0

**Table 11:** *R*_*v*_ for *n* = 2*ζ* + 1 and *t* = 2*ϖ*. For 15 ≤ *n* ≤ 21, $\left\lfloor \frac{\zeta }{4}\right\rfloor +2\leq \varpi \leq \left\lfloor \frac{2\zeta -1}{4}\right\rfloor$ and for *n* ≥ 23, $\left\lfloor \frac{\zeta }{4}\right\rfloor +3\leq \varpi \leq \left\lfloor \frac{2\zeta -1}{4}\right\rfloor$.

Distance of vertices from	Ψ_1_	Ψ_2_	Ψ_3_	Ψ_4_
*v*_*χ*_(1 ≤ *χ* ≤ *ϖ*)	0	*ζ* − 2*ϖ* + *χ*	*ζ* − 2*ϖ* + *χ*	*χ*
*v*_*ϖ*+1_	0	*ζ* − *ϖ*	*ζ* − *ϖ* + 1	*ϖ* + 1
*v*_*ϖ*+*χ*+1_(1 ≤ *χ* ≤ *ϖ*)	0	*ζ* − *ϖ* − *χ*	*ζ* − *ϖ* − *χ* + 1	*ϖ* − *χ* + 2
*v*_2*ϖ*+*χ*−1_(3 ≤ *χ* ≤ *ζ* − 2*ϖ* + 1)	0	*ζ* − 2*ϖ* − *χ* + 2	*ζ* − 2*ϖ* − *χ* + 3	*χ*
*v*_*ζ*+1_	1	0	1	*ζ* − 2*ϖ* + 2
$v_{2\varpi +\chi -1}\left(\zeta -2\varpi +3\leq \chi \leq \;\left\lceil \frac{\zeta }{2}\right\rceil +1\right)$	*χ* − *ζ* + 2*ϖ* − 1	*χ* − *ζ* + 2*ϖ* − 2	0	*χ*
$v_{\zeta +\chi }\left(2\varpi -\left\lfloor \frac{\zeta }{2}\right\rfloor +1\leq \chi \leq \;\left\lfloor \frac{\zeta }{2}\right\rfloor +1\right)$	*χ*	*χ* − 1	0	2*ϖ* − *χ* + 2
$v_{2\zeta -\chi +2}\left(\varpi \leq \chi \leq \;\left\lceil \frac{\zeta }{2}\right\rceil \right)$	*χ*	*ζ* − *χ* + 1	0	2*ϖ* − *ζ* + *χ*
*v*_*ζ*+2*ϖ*−*χ*+2_(1 ≤ *χ* ≤ 3*ϖ* − *ζ* − 1)	*ζ* − 2*ϖ* + *χ*	2*ζ* − 4*ϖ* + *χ* + 1	0	*χ*
*v*_2*ζ*−*χ*+2_(1 ≤ *χ* ≤ *ζ* − 2*ϖ*)	*χ*	*ζ* − 2*ϖ* + *χ* + 1	*ζ* − 2*ϖ* − *χ* + 1	0

**Table 12:** *R*_*v*_ for *n* = 2*ζ* + 1 and *t* = 2*ϖ* − 1 where $2\leq \varpi \leq \lfloor \frac{2\zeta +5}{8}\rfloor$.

Distance of vertices from	Ψ_1_	Ψ_2_	Ψ_3_	Ψ_4_
*v*_*χ*_(1 ≤ *χ* ≤ *ϖ*)	0	*ζ* − 2*ϖ* + *χ* + 1	*ζ* − 2*ϖ* + *χ* + 1	*χ*
*v*_*ϖ*+*χ*_(1 ≤ *χ* ≤ *ϖ*)	0	*ζ* − *ϖ* − *χ* + 1	*ζ* − *ϖ* − *χ* + 2	*ϖ* − *χ* + 2
$v_{2\varpi +\chi -2}\left(3\leq \chi \leq \left\lfloor \frac{\zeta }{2}\right\rfloor +1\right)$	0	*ζ* − 2*ϖ* − *χ* + 3	*ζ* − 2*ϖ* − *χ* + 4	*χ*
$v_{\zeta -\chi +1}\left(1\leq \chi \leq \;\left\lceil \frac{\zeta }{2}\right\rceil -2\varpi +1\right)$	0	*χ*	*χ* + 1	2*ϖ* + *χ*
*v*_*ζ*+1_	1	0	1	2*ϖ*
*v*_*ζ*+*χ*+1_(1 ≤ *χ* ≤ 2*ϖ* − 1)	*χ* + 1	*χ*	0	2*ϖ* − *χ*
$v_{\zeta +2\varpi +\chi }\left(1\leq \chi \leq \;\left\lceil \frac{\zeta }{2}\right\rceil -2\varpi +1\right)$	2*ϖ* + *χ*	2*ϖ* + *χ* − 1	*χ*	0
$v_{2\zeta -\chi +2}\left(\varpi \leq \chi \leq \left\lceil \frac{\zeta }{2}\right\rceil \right)$	*χ*	*ζ* − *χ* + 1	*ζ* − *χ* − 2*ϖ* + 2	0
*v*_2*ζ*−*ϖ*+*χ*+2_(1 ≤ *χ* ≤ *ϖ* − 1)	*ϖ* − *χ*	*ζ* − *ϖ* − *χ* + 2	*ζ* − 3*ϖ* + *χ* + 2	0

**Table 13:** *R*_*v*_ for *n* = 2*ζ* + 1 and *t* = 2*ϖ* − 1. For 9 ≤ *n* ≤ 33, $\left\lfloor \frac{2\zeta +5}{8}\right\rfloor +1\leq \varpi \leq \left\lfloor \frac{2\zeta +5}{8}\right\rfloor +1$ and for *n* ≥ 35, $\left\lfloor \frac{2\zeta +5}{8}\right\rfloor +1\leq \varpi \leq \left\lfloor \frac{2\zeta +5}{8}\right\rfloor +2$.

Distance of vertices from	Ψ_1_	Ψ_2_	Ψ_3_	Ψ_4_
*v*_*χ*_(1 ≤ *χ* ≤ *ϖ*)	0	*ζ* − 2*ϖ* + *χ* + 1	*ζ* − 2*ϖ* + *χ* + 1	*χ*
*v*_*ϖ*+*χ*_(1 ≤ *χ* ≤ *ϖ*)	0	*ζ* − *ϖ* − *χ* + 1	*ζ* − *ϖ* − *χ* + 2	*ϖ* − *χ* + 2
*v*_2*ϖ*+*χ*−2_(3 ≤ *χ* ≤ *ζ* − 2*ϖ* + 2)	0	*ζ* − 2*ϖ* − *χ* + 3	*ζ* − 2*ϖ* − *χ* + 4	*χ*
*v*_*ζ*+1_	1	0	1	*ζ* − 2*ϖ* + 3
$v_{2\varpi +\chi -2}\left(h-2\varpi +4\leq \chi \leq \;\left\lceil \frac{\zeta }{2}\right\rceil +1\right)$	*χ* − *ζ* + 2*ϖ* − 2	*χ* − *ζ* + 2*ϖ* − 3	0	*χ*
$v_{\zeta +\chi }\left(2\varpi -\left\lfloor \frac{\zeta }{2}\right\rfloor \leq \chi \leq \;\left\lfloor \frac{\zeta }{2}\right\rfloor +1\right)$	*χ*	*χ* − 1	0	2*ϖ* − *χ* + 1
$v_{\zeta +2\varpi -\chi +1}\left(1\leq \chi \leq \;2\varpi -\left\lfloor \frac{\zeta }{2}\right\rfloor -1\right)$	*ζ* − 2*ϖ* + *χ* + 1	2*ϖ* − *χ*	0	*χ*
*v*_*ζ*+2*ϖ*+*χ*_(1 ≤ *χ* ≤ *ζ* − 3*ϖ* + 2)	*ζ* − 2*ϖ* − *χ* + 2	2*ϖ* + *χ* − 1	*χ*	0
*v*_2*ζ*−*χ*+2_(1 ≤ *χ* ≤ *ϖ* − 1)	*χ*	*ζ* − 2*ϖ* + *χ* + 2	*h* − 2*ϖ* − *χ* + 2	0

**Table 14:** *R*_*v*_ for *n* = 2*ζ* + 1 and *t* = 2*ϖ* − 1. For 17 ≤ *n* ≤ 33, $\left\lfloor \frac{2\zeta +5}{8}\right\rfloor +2\leq \varpi \leq \left\lfloor \frac{\zeta }{2}\right\rfloor$ and for *n* ≥ 35, $\left\lfloor \frac{2\zeta +5}{8}\right\rfloor +3\leq \varpi \leq \left\lfloor \frac{\zeta }{2}\right\rfloor$.

Distance of vertices from	Ψ_1_	Ψ_2_	Ψ_3_	Ψ_4_
*v*_*χ*_(1 ≤ *χ* ≤ *ϖ*)	0	*ζ* − 2*ϖ* + *χ* + 1	*ζ* − 2*ϖ* + *χ* + 1	*χ*
*v*_*ϖ*+*χ*_(1 ≤ *χ* ≤ *ϖ*)	0	*ζ* − *ϖ* − *χ* + 1	*ζ* − *ϖ* − *χ* + 2	*ϖ* − *χ* + 2
*v*_2*ϖ*+*χ*−2_(3 ≤ *χ* ≤ *ζ* − 2*ϖ* + 2)	0	*ζ* − 2*ϖ* − *χ* + 3	*ζ* − 2*ϖ* − *χ* + 4	*χ*
*v*_*ζ*+1_	1	0	1	*ζ* − 2*ϖ* + 3
$v_{2\varpi +\chi -2}\left(\zeta -2\varpi +4\leq \chi \leq \;\left\lceil \frac{\zeta }{2}\right\rceil +1\right)$	*χ* − *ζ* + 2*ϖ* − 2	*χ* − *ζ* + 2*ϖ* − 3	0	*χ*
$v_{\zeta +\chi }\left(2\varpi -\left\lfloor \frac{\zeta }{2}\right\rfloor \leq \chi \leq \;\left\lfloor \frac{\zeta }{2}\right\rfloor +1\right)$	*χ*	*χ* − 1	0	2*ϖ* − *χ* + 1
$v_{2\zeta -\chi +2}\left(\varpi \leq \chi \leq \;\left\lceil \frac{\zeta }{2}\right\rceil \right)$	*χ*	*ζ* − *χ* + 1	0	2*ϖ* − *ζ* + *χ* − 1
*v*_*ζ*+2*ϖ*−*χ*+1_(1 ≤ *χ* ≤ 3*ϖ* − *ζ* − 2)	*ζ* − 2*ϖ* + *χ* + 1	2*ζ* − 4*ϖ* + *χ* + 3	0	*χ*
*v*_2*ζ*−*χ*+2_(1 ≤ *χ* ≤ *ζ* − 2*ϖ* + 1)	*χ*	*ζ* − 2*ϖ* + *χ* + 2	*ζ* − 2*ϖ* − *χ* + 2	0

**Table 15:** *R*_*v*_ for *n* = 2*ζ* + 1 and *t* = *ζ* = 2*ϖ*.

Distance of vertices from	Ψ_1_	Ψ_2_	Ψ_3_	Ψ_4_
*v*_*χ*_(1 ≤ *χ* ≤ *ϖ*)	0	*χ* + 1	*χ* + 1	*χ*
*v*_*ϖ*+1_	0	*ϖ* + 1	*ϖ* + 2	*ϖ* + 1
*v*_*ϖ*+*χ*+1_(1 ≤ *χ* ≤ *ϖ*)	0	*ζ* − *ϖ* − *χ* + 1	*ζ* − *ϖ* − *χ* + 2	*ζ* − *ϖ* − *χ* + 2
*v*_*ζ*+2_	1	0	1	3
*v*_*ζ*+*χ*+2_(1 ≤ *χ* ≤ *ϖ* − 2)	*χ* + 1	*χ*	0	*χ* + 3
*v*_*ζ*+*ϖ*+1_	*ϖ*	*ϖ* − 1	0	*ϖ*
*v*_*ζ*+*ϖ*+2_	*ϖ*	*ϖ*	0	*ϖ* − 1
*v*_*ζ*+*ϖ*+*χ*+2_(1 ≤ *χ* ≤ *ϖ* − 2)	*ζ* − *ϖ* − *χ*	*ζ* − *ϖ* − *χ* + 2	0	*ζ* − *ϖ* − *χ* − 1
*v*_2*ζ*+1_	1	3	1	0

**Table 16:** *R*_*v*_ for *n* = 2*ζ* + 1 and *t* = *ζ* = 2*ϖ* − 1.

Distance of vertices from	Ψ_1_	Ψ_2_	Ψ_3_	Ψ_4_
*v*_*χ*_(1 ≤ *χ* ≤ *ϖ*)	0	*χ* + 1	*χ* + 1	*χ*
*v*_*ϖ*+*χ*_(1 ≤ *χ* ≤ *ϖ*)	0	*h* − *ϖ* − *χ* + 2	*ζ* − *ϖ* − *χ* + 3	*ζ* − *ϖ* − *χ* + 3
*v*_5_ for *n* = 7	1	0	1	2
*v*_6_ for *n* = 7	2	1	0	1
*v*_7_ for *n* = 7	1	2	1	0
*v*_*ζ*+2_ for *n* ≥ 9	1	0	1	3
*v*_*ζ* + *χ*+2_(1 ≤ *χ* ≤ *ϖ* − 3) for *n* ≥ 9	*χ* + 1	*χ*	0	*χ* + 3
*v*_*ζ*+*ϖ*+*χ*−1_(1 ≤ *χ* ≤ 2) for *n* ≥ 9	*ζ* − *ϖ* + *χ* − 1	*ζ* − *ϖ* + *χ* − 2	0	*ζ* − *ϖ* − *χ* + 2
*v*_*ζ*+*ϖ*+2_ for *n* ≥ 9	*ϖ* − 1	*ϖ*	0	*ϖ* − 2
*v*_*ζ*+*ϖ*+*χ*_(1 ≤ *χ* ≤ *ϖ* − 3) for *n* ≥ 9	*ζ* − *ϖ* − *χ*	*ζ* − *ϖ* − *χ* + 2	0	*ζ* − *ϖ* − *χ* − 1
*v*_2*ζ*+1_ for *n* ≥ 9	1	3	1	0

Now, we establish that $P(C_{n}^{t})\geq 4$. To do this, we demonstrate that $P(C_{n}^{t})\neq 3$. Suppose to the contrary that $P(C_{n}^{t})=3$. Let Ψ = {Ψ_1_, Ψ_2_, Ψ_3_} be a FT resolving partition of $C_{n}^{t}$. Let *v*_*i*_ ∈ Ψ_1_ and *N*(*v*_*i*_) = {*u*_1_, *u*_2_, *u*_3_}, where *u*_3_ is also a vertex of degree 3. Suppose that |Ψ_1_| = 1, and *N*(*v*_*i*_) ⊆ Ψ_2_ ∪ Ψ_3_, by Pigeonhole principle |*N*(*v*_*i*_) ∩ Ψ_2_| ≥ 2 or |*N*(*v*_*i*_) ∩ Ψ_3_| ≥ 2. Without loss of generality, we assume that at least two vertices *a*, *b* ∈ *N*(*v*_*i*_) ∩ Ψ_2_. Since, *r*(*a*|Ψ) = (1, 0, *c*_1_) and *r*(*b*|Ψ) = (1, 0, *c*_2_) show equal distances at two positions, resulting in a contradiction.

Now considering that |Ψ_1_| ≥ 2 and *v*_*i*_ ∈ Ψ_1_, we encounter the following scenarios:

**Case 1:** If each of the three vertices *u*_1_, *u*_2_, *u*_3_ ∈ Ψ_1_, then *r*(*v*_*i*_|Ψ) = (0, *p*_0_, *q*_0_), *r*(*u*_1_|Ψ) = (0, *p*_1_, *q*_1_), *r*(*u*_2_|Ψ) = (0, *p*_2_, *q*_2_) and *r*(*u*_3_|Ψ) = (0, *p*_3_, *q*_3_). As *p*_0_ − 1 ≤ *p*_1_, *p*_2_, *p*_3_ ≤ *p*_0_ + 1, thus, two vertices having the same distance at two locations lead to a contradiction.
**Case 2:** If *r*(*v*_*i*_|Ψ) = (0, 1, *q*_0_), and two vertices *u*_1_, *u*_2_ ∈ Ψ_1_ and another vertex *u*_3_ ∈ Ψ_2_, then, *r*(*u*_1_|Ψ) = (0, *p*_1_, *q*_1_), *r*(*u*_2_|Ψ) = (0, *p*_2_, *q*_2_) and *r*(*u*_3_|Ψ) = (1, 0, *q*_3_). Since 1 ≤ *u*_1_, *u*_2_ ≤ 2, two vertices will again have the same distance at two positions, resulting in a contradiction.
**Case 3:** If one vertex say, *u*_1_ ∈ Ψ_1_, and two vertices *u*_2_, *u*_3_ ∈ Ψ_2_, then *r*(*v*_*i*_|Ψ) = (0, 1, *q*_0_), *r*(*u*_1_|Ψ) = (0, *p*_1_, *q*_1_), *r*(*u*_2_|Ψ) = (1, 0, *q*_2_) and *r*(*u*_3_|Ψ) = (1, 0, *q*_3_). Again *r*(*u*_2_|Ψ) and *r*(*u*_3_|Ψ) are the same at two positions, resulting in a contradiction.
**Case 4:** If *N*(*v*_*i*_) ∩ Ψ_1_ = ∅, all the three vertices *u*_1_, *u*_2_, *u*_3_ ∈ Ψ_2_, then *r*(*v*_*i*_|Ψ) = (0, 1, *q*_0_), *r*(*u*_1_|Ψ) = (1, 0, *q*_1_), *r*(*u*_2_|Ψ) = (1, 0, *q*_2_), *r*(*u*_3_|Ψ) = (1, 0, *q*_3_). Again *r*(*u*_1_|Ψ), *r*(*u*_2_|Ψ) and *r*(*u*_3_|Ψ) are the same at two positions, resulting in a contradiction.
**Case 5:** If *N*(*v*_*i*_) ∩ Ψ_1_ = ∅, two vertices *u*_1_, *u*_2_ ∈ Ψ_2_, and one vertex *u*_3_ ∈ Ψ_3_, then *r*(*v*_*i*_|Ψ) = (0, 1, *q*_0_), *r*(*u*_1_|Ψ) = (1, 0, *q*_1_), *r*(*u*_2_|Ψ) = (1, 0, *q*_2_). Again *r*(*u*_1_|Ψ) and *r*(*u*_2_|Ψ) are the same at two positions, resulting in a contradiction.
**Case 6:** If one vertex say, *u*_1_ ∈ Ψ_1_, *u*_2_ ∈ Ψ_2_ and *u*_3_ ∈ Ψ_3_, then *r*(*v*_*i*_|Ψ) = (0, 1, 1). We have the following subcases:
  **Case 6(a)** If *t* = 2, then *r*(*u*_1_|Ψ) = (0, 2, 1), resulting in a contradiction.
  **Case 6(b)** If *t* = 3, then consider *N*(*u*_1_) = {*w*_1_, *v*_*i*_}, *N*(*u*_2_) = {*w*_2_, *v*_*i*_} and *N*(*u*_3_) = {*w*_3_, *v*_*i*_}. Let *w*_1_ ∈ Ψ_1_ and *w*_2_, *w*_3_ ∈ Ψ_2_ ∪ Ψ_3_. We can assume without loss of generality that *w*_2_, *w*_3_ ∈ Ψ_2_, then *r*(*u*_2_|Ψ) = (1, 0, 2), *r*(*w*_2_|Ψ) = (2, 0, *c*_1_) and *r*(*w*_3_|Ψ) = (2, 0, 1). The representation of two vertices being identical at two positions leads to a contradiction. If *w*_3_ ∈ Ψ_2_ and *w*_2_ ∈ Ψ_3_, then, *r*(*u*_2_|Ψ) = (1, 0, 1) and *r*(*w*_3_|Ψ) = (2, 0, 1), results to a contradiction. Now if *w*_2_ ∈ Ψ_2_ and *w*_3_ ∈ Ψ_3_, then, *r*(*u*_1_|Ψ) = (0, 2, 2) and *r*(*w*_1_|Ψ) = (0, *c*_2_, 1). As *r*(*v*_*i*_|Ψ) and *r*(*w*_1_|Ψ) are identical at two positions which is again a contradiction.
  **Case 6(c)** For $4\leq t\leq \lfloor \frac{n}{2}\rfloor$, consider *N*(*u*_1_) = {*w*_1_, *v*_*i*_}, *N*(*u*_2_) = {*w*_2_, *v*_*i*_} and $N\left(u_{3}\right)=\left\{w_{3},w_{3}^{\prime },v_{i}\right\}$. Let *w*_1_ ∈ Ψ_1_ and $w_{2},w_{3},w_{3}^{\prime }\in \Psi_{2}\cup \Psi_{3}$. We can assume without loss of generality that $w_{2},w_{3},w_{3}^{\prime }$ belong to Ψ_2_, so, *r*(*u*_2_|Ψ) = (1, 0, *d*_1_), *r*(*w*_2_|Ψ) = (2, 0, *d*_2_) and *r*(*w*_3_|Ψ) = (2, 0, 1). Since the representation of *w*_2_ and *w*_3_ at two positions are identical, thus a contradiction. Now if $w_{3},w_{3}^{\prime }\in \Psi_{2}$ and *w*_2_ ∈ Ψ_3_, *r*(*u*_2_|Ψ) = (1, 0, 1) and *r*(*w*_3_|Ψ) = (2, 0, 1). Since the representation of two vertices is identical at two positions, this leads to a contradiction. If *w*_2_ ∈ Ψ_2_ and $w_{3},w_{3}^{\prime }\in \Psi_{3}$, then, *r*(*u*_3_|Ψ) = (1, 2, 0), *r*(*w*_3_|Ψ) = (2, *f*_1_, 0) and $r\left(w_{3}^{\prime }|\Psi \right)=\left(2,f_{2},0\right)$. Two vertices have the same distance at two positions, resulting in a contradiction.

Based on all the aforementioned cases, it is evident that $P(C_{n}^{t})\geq 4$, thereby concluding the proof.

## Conclusion

In this paper, we have demonstrated that the fault-tolerant partition dimension (FTPD) of a cycle with chord graph $C_{n}^{t}$ is $P(C_{n}^{t})=4$ for *n* ≥ 4 and $2\leq t\leq \lfloor \frac{n}{2}\rfloor$. This result indicates that the FTPD of these graphs is constant, specifically 4, regardless of the values of *n* and *t* within the specified ranges. Our findings contribute to a deeper understanding of the FTPD in the context of the cycle with chord graphs, providing a clear and consistent value for this parameter across various graph sizes and chord configurations.

Despite this advancement, several questions remain unresolved in the field. One particularly intriguing open problem is to determine whether similar constant values can be established for the FTPD of other classes of graphs or under different graph parameters. Further exploration is needed to generalize the results and understand the implications of FTPD in broader contexts. We encourage future research to investigate these aspects and explore potential applications of these findings in network design, optimization, and fault tolerance. Moreover, we propose the following open problem.

**Open Problem 0.7** *Compute FTPD of multilevel-cycles with chord*.

**Open Problem 0.8** *Compute fault tolerant metric dimension of the cycle with chord*.
